# Examining gender as an issue of policy priority: a case study of four Kenyan health policy implementation strategies

**DOI:** 10.3389/fpubh.2025.1615792

**Published:** 2025-07-31

**Authors:** Henry Owoko Odero, Doris Kwesiga, Sally Odunga, Damazo T. Kadengye, Sylvia Kiwuwa-Muyingo

**Affiliations:** African Population and Health Research Center (APHRC), Data Synergy and Evaluation Unit, Nairobi, Kenya

**Keywords:** gender equity, health policy, policy prioritization, Kenya, policy analysis

## Abstract

**Background:**

Despite global and national commitments to gender equity, including Sustainable Development Goal 5, health policy processes often fail to adequately integrate gender considerations. This study explores how gender is prioritized in the development and implementation of four Kenyan Health Policy Implementation Strategies (HPIS), offering insight into the role of institutional actors, policy framing, and contextual factors.

**Methods:**

A qualitative case study was conducted involving content analysis of four HPIS documents - KASF II 2020, NSP-NCD 2021, NSP-TB 2019, and COVID-TTS 2019 - and semi-structured interviews with 16 policy stakeholders. Data were analyzed using the Shiffman and Smith framework, which examines actors, ideas, policy environments, and issue characteristics influencing policy prioritization.

**Results:**

Gender prioritization varied among the policies under review. In the KASF II 2020, strong leadership from organizations such as the National AIDS Control Council (NACC), UNAIDS, and UNDP effectively drove gender integration. These actors successfully advocated for gender-responsive metrics and policies, supported by robust gender-disaggregated data. The policy framing (ideas) clearly recognized HIV as inherently gendered, emphasizing differentiated impacts across gender groups. The favorable policy environment, including alignment with Kenya’s Universal Health Coverage under the “Big Four Agenda,” enabled gender mainstreaming. Conversely, the NSP-NCD 2021 acknowledged gender primarily through a vulnerability lens, identifying gender-specific behaviors such as higher tobacco use among men. However, outdated data and limited structural guidance restricted the practical application of gender-specific strategies. The NSP-TB 2019 strategy on the other hand exhibited minimal gender integration due to the absence of dedicated gender advocacy actors and ambiguity in policy framing regarding gender as a social versus biological issue. The COVID-TTS was rapidly developed in response to the pandemic emergency, initially neglecting gender considerations entirely. Later, as gender disparities became apparent, the policy environment shifted slightly toward acknowledging these disparities, but actions remained largely reactive and inconsistent.

**Conclusion:**

Effective gender integration in Kenyan health policies depended significantly on active leadership by key actors, strategic framing of gender issues, a conducive policy environment, and reliable gender-specific data. Strengthening these elements is key in improving future gender responsiveness in health policy.

## Highlights

- Gender integration in Kenyan health policies is inconsistent, with meaningful inclusion evident only in HIV-focused strategies like KASF II 2020.
- Policy actor leadership, particularly by institutions like NACC, plays a critical role in embedding gender considerations during health policy development.
- Emergency responses, such as COVID-TTS 2019, often sideline gender concerns, highlighting the need for pre-existing equity frameworks.
- Weak gender-disaggregated data and lack of implementation guidance hinders the operationalization of gender mainstreaming in health strategies.
- Public health implication: Strengthening institutional mechanisms and data systems for gender responsive planning is essential for equitable and effective health policy implementation.

## Introduction

Health professionals, researchers, and policymakers increasingly recognize the implications of gender-based inequities in driving health outcomes (1, 2). The 2015 United Nations-Sustainable Development Goal 5 (UN-SDG 5) presents a strategic opportunity for the global health community to advance sex and gender equality by addressing the systemic challenges faced by individuals (3).

Contemporary studies, define gender as socially constructed roles, behaviors, and norms associated with being a man, woman, or other identities (4, 5), while sex is a biological construct encompassing anatomical and physiological differences (6). The distinction between sex and gender are foundational and important and have been covered extensively elsewhere (7, 8).

Sex and gender significantly impact clinical and public health practice by influencing disease prevention, diagnosis, treatment, and outcomes (9, 10). For instance, gender affects access to care and the standard of care received, both at the individual and population level, often as a result of various institutional and structural obstacles, including sexism, overt discrimination, and implicit bias (11). Existing evidence shows a generalized female advantage for indicators such as life expectancy and a generalized male advantage for indicators such as years lived with chronic disabilities (12, 13). This is because, Sex and gender are embedded within a matrix of intersecting social categories such as class, geography, race, and socio-political status (14). Kimberlé Crenshaw’s, in their seminal concept of intersectionality, emphasizes how these overlapping identities create unique forms of discrimination that cannot be explained by any single axis alone (15). The concept of intersectionality demands attention to how interacting power structures shape lived experiences in health, with race-class-gender intersections often amplifying disadvantage. This has been described by Deborah King as “multiple jeopardy,” where she teases out the compounding rather than merely additive effects of overlapping oppression (16).

In public health policy scholarship, intersectionality has been increasingly applied to show how policy neglect rooted in narrow categorizations can perpetuate inequities; for example, Olena Hankivsky et al. demonstrate that intersectionality-based policy analysis foregrounds dimensions like history, lived experience, and diverse knowledges, enabling equity-driven policy solutions not captured by more traditional frameworks (14, 17).

Evidence from employment and economic sectors have demonstrated that gender consideration during policy formulation leads to equitable outcomes (18), yet governments’ policy responses to health continue to pay very little and inconsistent consideration (1).

In this study, we critically examine how gender was afforded priority during the development of four Kenyan Health Policy Implementation Strategies (HPIS). To systematically analyze this, we apply Shiffman and Smith’s policy prioritization framework (19), a robust analytical framework that conceptualizes policy prioritization, through four interrelated domains: actor power (including leadership, institutional cohesion, and civil society engagement), ideas (encompassing both internal consensus and external framing), political context (such as policy windows and global governance structures), and issue characteristics (including measurable indicators, perceived severity, and availability of effective interventions) (20). Using this framework, we explore how these domains collectively enable or constrain the inclusion of gender considerations in policy design and implementation.

Understanding the policy prioritization process is important in interpreting why, despite widespread normative and international commitments to gender equity, attention to gender remains inconsistent across health policy domains (21). For instance, strong actor coalitions and effective framing may elevate gender in one policy but not another (22). Equally, absence of credible indicators or closed policy windows can marginalize gender, even within ostensibly progressive mandates (23). By mapping these interactions, our study generates insights into the institutional, discursive, and contextual factors that shape Kenya’s gender responsiveness in health policymaking.

The process of policy prioritization has been studied in various contexts, such as drowning prevention in Bangladesh (24), urban health among urban poor populations in low-income countries (25) and government’s attentiveness to citizens’ political Priorities in the United States of America (26). These studies have demonstrated that policy priority is influenced by a myriad of factors, including socio-political context, public opinion, donor interests, and advocacy efforts (25, 26).

The current scholarship on policy prioritization has predominantly concentrated on policy makers at the parliamentary and ministerial level who are either elected or appointed, therefore inadequately accounting for the role of non-elected, and non-appointed, career civil servants, otherwise referred to as bureaucrat (27). Empirical evidence indicates that bureaucrats play a crucial role in the policy process due to their permanence and expertise, contrasting with the transient nature of elected politicians (28, 29). Thus, a comprehensive understanding of policy prioritization in democracies like Kenya requires an examination that includes the contributions of bureaucrats who often remain faceless but integral to governance and policy continuity.

In this study, we employed a qualitative case study design, to examine gender prioritization in health policy, within its real-world context (30). We conducted a document content analysis of four Kenyan health policy implementation strategy (HPIS) using a structured extraction matrix aligned with Shiffman and Smith’s four policy prioritization domains (actors, ideas, political context, issue characteristics) (19). Concurrently, we undertook semi-structured key informant interviews with purposively sampled stakeholders, including career civil servants, policy analysts, and representatives from non-governmental organizations. Interviewees were selected based on their direct involvement in policy development or implementation, following purposive and snowball sampling. Interviews were recorded, transcribed verbatim, and analyzed using a thematic codebook developed deductively from Shiffman and Smith’s framework and refined inductively during iterative coding cycles. We integrated both document and interview data to identify cross-policy patterns and divergences in how gender was framed, institutionalized, and operationalized.

## Materials and methods

### Study design

This study took place in two main stages. First, we selected four disease areas where gender differences are well documented, especially in how men and women experience the disease- from risk exposure all the way through to treatment outcomes (31, 32). Based on this, we identified four health policy implementation strategies (HPIS) for analysis: the Kenya AIDS Strategic Framework 2020/21–2024/25 (KASF II 2020), the National Strategic Plan for the Prevention and Control of Non-Communicable Diseases 2021/22 (NSP-NCD 2021), the National Strategic Plan for Tuberculosis, Leprosy and Lung Health 2019–2023 (NSP-TB 2019), and the Targeted Testing Strategy for COVID-19 in Kenya (COVID-TTS 2019). We focused on policies developed from 2018 onwards to reduce the effects of recall bias and to ensure that the key individuals involved in policy formulation were still available and accessible for interviews.

### Sampling and data collection

We identified 57 potential stakeholders involved in the development of the selected policies. Using a combination of contributor lists from policy documents and snowball sampling, we reached out to these individuals. In total, we conducted 16 key informant interviews. Each policy had five respondents, except COVID-TTS 2019, where only one interview was possible, a notable limitation. The participants included nine representatives from government institutions and seven from non-governmental organizations, civil society, and the private sector. Alongside the interviews, we conducted a document analysis of the four policy texts to complement and crosscheck insights gathered from the interviews (Tables 1, 2).

**Table 1:** Key informants by type of organization.

Type of respondent	Interviewed
Government	9
Other actors (NGO, CSO and Consultants)	7

**Table 2:** Distribution of interviews by health policy implementation strategies.

Policy implementation strategy	Short name	Disease area	Number contacted	Number interviewed
National Strategic Plan for Tuberculosis, Leprosy and Lung Health 2019–2023	NSP-TB 2019	Tuberculosis	15	5
Targeted Testing Strategy for Corona Virus Disease 2019 (COVID-19)	COVID TTS 2019	COVID-19	16	1
Kenya AIDS Strategic Framework (KASF) II 2020/21–2024/25	KASF II 2020	HIV	19	5
National strategic plan for the prevention and control of non-communicable diseases 2021/22–2025/26	NSP-NCD 2021	Diabetes and hypertension	12	5

Although we conducted six key informant interviews across the other HPIS cases, only a single interview was obtained for the COVID-TTS strategy due to lack of willingness to participate by those involved in developing the COVID-TTS. We acknowledge that this limits the depth of perspective on this policy and weakens triangulation. Nonetheless, we retained the case in order to reflect the unique and time-sensitive considerations of public health policymaking under crisis conditions.

### Analytical framework

Our analysis was guided by the Shiffman and Smith framework (19) (Table 3), which helped in understanding why certain health issues receive political attention over others. This framework considers four main dimensions that influence policy prioritization: actors, ideas, the policy environment, and issue characteristics. It has been widely used in health policy research and provided a solid structure for our data analysis.

**Table 3:** Shiffman and Smith framework.

Category	Category definition by Shiffman and Smith (19)	Category definition in this study
Actor	Policy community cohesion: The degree of cohesion among stakeholders involved in development of the policy implementation strategy.	Identify the institutional and individual actors involved in the policy development process and their motivations.
	Leadership: The presence of individuals capable of raising awareness on gendered health differences and mobilizing resources.	
	Guiding institutions: The effectiveness of organizations or coordinating mechanisms with a mandate to lead the development of the implementation strategy.	
	Civil society mobilization: The extent to which ‘gender organizations have mobilized the necessary support from international and national political authorities.	
Ideas	Internal frame: The way policy community agree on the definition of, causes of, and solutions to gendered health as an issue	Assessed whether the policy acknowledged gendered health as an issue and how these were portrayed within the policy framework.
	External frame: The way to portray gendered health as an issue outside of the policy community	
Policy environment	Policy window: Political momentum when global community favorably align with gendered health as an issue	Examined the context in which the policy was formulated, including socio-political and economic factors that may have influenced the development and implementation of the policy.
	Governance structure: The degree to which norms and institutions operating in the sector provide a platform for effective collective action	
Issue characteristics	Credible indicators: Clear and measurable indicators which can trace the severity and progress on gender differences in health.	Evaluated the policy’s use of credible indicators to track health outcomes and determine if the policy adequately highlighted the severity of gendered health components. We also assessed whether the policy proposed effective interventions to address gendered health disparities.
	Severity: The size of the burden relative to other issues (i.e., mortality)	
	Effective interventions: The extent to which proposed means of addressing the problem are clearly explained, cost-effective, evidence-based, easy to implement and affordable.	

In this study, ‘gender integration’ is operationalized by (i) explicit inclusion of gender-disaggregated indicators; (ii) designated institutional leadership or roles for gender mainstreaming; and (iii) strategic framing of gender as a structural driver of health inequity. We coded policies as ‘strong’ or ‘weak’ in gender integration based on the presence or absence of these elements. This guided our coding and is presented in Table 4.

**Table 4:** Gender integration coding framework.

Dimension	Definition	Coding rule	Strength classification
1. Gender-disaggregated indicators	Presence of sex/gender-specific indicators or targets for monitoring policy outcomes	Coded ‘Strong’ if ≥1 indicator explicitly disaggregated by sex/gender is included in the policy	Strong = ≥1 indicator Weak = none
2. Institutional leadership or coordination	Presence of a designated office, focal person, or institutional structure responsible for gender integration	Coded ‘Strong’ if such a role is formally assigned or referenced in the policy	Strong = role specified Weak = no role assigned
3. Structural framing of gender	Framing of gender as a structural or systemic determinant of health inequities	Coded ‘Strong’ if gender is linked to social determinants, norms, or power structures	Strong = structural framing present Weak = absent or purely demographic framing

### Data analysis

We began by assessing policy document strength on gender consideration (Supplementary Table 1). We then reviewed each policy document to extract both clear and subtle references to gender (Supplementary Table 2). Semi-structured interviews were then used to explore how gender issues were discussed, framed, or sidelined during policy development. The interview guide was designed to probe the roles of key actors, the framing of gender-related ideas, the influence of the broader policy environment, and how the nature of the health issue itself shaped policy choices (Supplementary Table 3).

Data were analyzed thematically (33) using initial codes based on the Shiffman and Smith framework. These codes were refined iteratively as we engaged more deeply with the data. To ensure clarity while maintaining fidelity to participants’ perspectives, we transcribed all interviews using clean verbatim methods (34) - meaning we preserved the core content but removed filler words and repetitions. For confidentiality, each interviewee was assigned a unique identifier in the reporting of findings.

## Results

### Health policy development in a changing landscape

Between 2015 and 2021, Kenya’s health policy environment evolved in response to shifting global norms and domestic political priorities. The development of four key policy strategies—KASF II 2020, NSP-NCD 2021, NSP-TB 2019, and COVID-TTS 2019—reflected both the momentum behind Universal Health Coverage (UHC) and the influence of the national “Big Four Agenda,” which prioritized healthcare reform. These strategies offered entry points for integrating gender, but such integration varied widely across policies and was often treated as secondary to broader goals such as disease burden reduction or emergency containment.

### Actors and leadership

Leadership by the National AIDS Control Council (NACC), UNAIDS, and UNDP was essential in embedding gender within KASF II. These actors helped institutionalize gender-responsive strategies and ensured gender-disaggregated indicators were included in the policy’s monitoring framework. In contrast, the NSP-TB development process lacked similar advocacy. Despite broad stakeholder representation, gender concerns were absent, in part due to the limited involvement of gender-focused civil society organizations. One participant plainly noted, *“We had everyone at the table… but gender never came up as a specific issue.” (Participant 9)*

In the NSP-NCD 2021 process, civil society groups such as Plan International and the NCD Alliance Kenya supported gender-inclusive language. However, participants emphasized that this inclusion was incidental, and not the result of a deliberate gender mainstreaming efforts. Meanwhile, the COVID-TTS process, driven by urgency, lacked a coherent actor network to advocate for gender, leading to its near-complete omission.

### Ideas and policy framing

The framing of gendered health as a policy issue was equally key in shaping its prioritization. HIV was widely understood as a gendered epidemic, which facilitated robust integration in KASF II. In contrast, TB and NCDs were framed in biomedical and lifestyle terms, respectively—narratives that did not naturally invite gender analysis. Participants in the NSP-TB process, for instance, debated whether gender as a social construct was even relevant, often conflating it with women’s issues, despite evidence of higher TB prevalence among men. In NSP-NCD, while male vulnerability to tobacco use was acknowledged, the policy fell short of proposing gender-specific responses.

COVID-TTS 2019 was shaped almost entirely by crisis discourse. Gender was not actively framed as relevant in initial drafts. Only later did policymakers begin to acknowledge gendered consequences—like rising domestic violence and maternal health disruptions—as the pandemic unfolded. As one participant stated, *“You are trying to implement and at the same time try to learn… you are not doing them in a perfect way.” (Participant 6)*

### Policy environment

The broader environment shaped policy opportunities for integrating gender. KASF II’s alignment with UHC and global HIV norms created a favorable backdrop for gender mainstreaming.

Similarly, international donor requirements and Kenya’s constitutional Two-Thirds Gender Rule kept gender on the agenda during the NSP-NCD process, even if substantive implementation frameworks were lacking. In contrast, NSP-TB and COVID-TTS were shaped by resource scarcity and political urgency, respectively—conditions that deprioritized gender.

For NSP-NCD, outdated data sources like the 2015 STEPWISE survey further weakened the evidence base for gender-responsive planning. The absence of current, disaggregated data meant that interventions were not targeted to specific vulnerabilities. One official explained, *“It has not given the how-to-do-it. It is upon the implementers… to come with different activities.” (Participant 15)*

### Issue characteristics

The nature of each health issue influenced how gender was addressed. HIV’s social, economic, and biological complexity—and its history of gender-focused advocacy—made it easier to justify gender-specific policies. KASF II benefited from decades of iterative learning, allowing for the institutionalization of gender norms. As one respondent put it, *“We are 38 years into the response… we have been able to improve and will do even better.” (Participant 9)*

NCDs and TB lacked this foundation. Gender was framed primarily through vulnerability and behavior in NSP-NCD, with no corresponding programmatic strategies. NSP-TB data showing men’s higher disease burden and women’s caregiving risks were not integrated into final plans. COVID-TTS, meanwhile, treated all populations as equally at risk, ignoring clear differences in access, exposure, and vulnerability. The crisis-response nature of this strategy limited opportunities for structured gender integration.

### Structural constraints and emerging concepts

Persistent structural barriers—particularly male-dominated leadership and inequitable resource allocation—were repeatedly cited as limiting gender mainstreaming. Several participants highlighted that women were often absent from decision-making spaces, especially those that control budgets: *“The people who allocate resources are often not women.” (Participant 14)*

KASF II was the only policy to explicitly acknowledge gender fluidity. However, its implementation faced institutional resistance, especially outside the health sector. Policymakers navigated this by framing inclusion under broader human rights and anti-discrimination narratives: *“We have been intentional… but in other sectors, if you do not fit within the norm, then it becomes an issue.” (Participant 11)*

Finally, gender-disaggregated data emerged as a foundational enabler of policy action. Participants noted that such data not only informed more responsive interventions but also empowered advocacy: *“If there is a certain intervention that is beneficial to women and scientific evidence shows that, then that will be picked on because it was evidence-based.” (Participant 7)*

## Discussion

This paper finds notable disparities in how gender is prioritized across the four Kenyan health policy implementation strategies, which it attributes to the differences in actor influence, idea framing, policy context, and the nature of the health issues. These elements are not isolated; rather, they often interact, shaping the extent to which gender is integrated into health policies, a relationship well-theorized in Shiffman and Smith’s framework (23).

The importance of powerful actor networks with a deliberate gender mandate cannot be overstated, especially their role in promoting gender as an issue of policy priority. The case of KASF II illustrates how the strategic involvement of well-positioned institutions such as NACC, with support from international agencies like UNAIDS and UNDP, can normalize gender responsiveness within policy community. This is consistent with findings across global health policy where sustained advocacy and institutional leadership have been instrumental in mainstreaming gender, particularly in HIV and reproductive health contexts (26, 27). In contrast, the lack of a unified actor groups and the lack of gender-oriented advocacy in the NSP-TB and COVID-TTS cases are what Shiffman describes as “dispersed coalitions” which are often unable to consolidate issue attention (23). This confirms Buse et al.’s assertion that policy neglect often results not from a lack of relevance but from the absence of coordinated actor engagement (28). However, actors alone cannot drive prioritization without persuasive framing. In this study, we see that gender-responsive HIV policies benefited from a long-standing recognition of HIV as a gendered epidemic. In contrast, biomedical and behavioral framings of TB and NCDs created conceptual blind spots, undermining intersectional analyses and reinforcing the notion of gender neutrality in disease response. These findings align with observations by Hawkes and Buse, who argue that technocratic framings often strip policy problems of their sociopolitical dimensions, thereby marginalizing equity considerations (29). Indeed, where gender is conflated with “women’s issues”—as seen in NSP-TB—there is limited traction for structural change, particularly in male-dominated policy forums. COVID-TTS exemplifies a different framing failure: the dominance of crisis discourse sidelined social vulnerability analysis entirely, reaffirming the critique that emergency responses often default to gender blindness unless gender is institutionally embedded beforehand (30).

The policy environment—shaped by timing, political incentives, data systems, and donor conditionalities—either constrains or enables gender integration. KASF II’s success can be partly attributed to alignment with UHC goals and global gender norms, illustrating how external pressure can foster compliance. Conversely, the COVID-TTS strategy, developed under conditions of political urgency and resource constraints, deprived gender advocates of procedural entry points. The importance of institutional timing and policy windows in advancing equity has been widely documented (31). Moreover, the NSP-NCD experience highlights how the absence of current, disaggregated data can dilute gender intent, as observed in other LMIC contexts where planning is often reactive and data-poor (32).

Importantly, the characteristics of the health issue itself shape gender prioritization. HIV, with its entrenched socio-behavioral complexity and historical global funding, provided a fertile ground for gendered engagement. TB and NCDs, by contrast, were framed around individual behavior and biomedical responses—approaches that rarely incentivize upstream gender analysis. This finding reinforces the view that disease narratives heavily mediate what kinds of solutions are seen as legitimate (33). COVID-19 further complicates this, as its initial technocratic response ignored gender differences in caregiving, employment, and exposure, until grassroots and media activism forced recognition of its gendered impacts (34).

Finally, structural constraints—notably gender imbalances in budgetary and leadership spaces— acted as persistent barriers to mainstreaming. That gender was most robustly addressed in KASF II, the only policy to institutionalize gender fluidity and non-binary inclusion, highlights how normative shifts are possible but remain fragile. As noted by Morgan et al., policies that challenge prevailing gender norms require not only institutional support but also protective mechanisms against rollback (35). Here, the role of gender-disaggregated evidence is Key, not merely for diagnostic purposes but as a tool of advocacy and accountability. When used strategically, such data can reframe policy debates, establish legitimacy for targeted interventions, and mobilize political will.

### Study limitations

As noted in methods, the reliance on one interview for COVID-TTS restricts the robustness of comparative insights and introduces possible bias in interpreting that case. While this restricts the depth of triangulation for that case, it also reflects real-world challenges of policy research.

## Conclusion

Taken together, this analysis suggests that gender prioritization in health policy is not solely a technical endeavor but a political act, shaped by who participates, how problems are defined, what resources are available, and how issues resonate within dominant political narratives. Efforts to strengthen gender integration must therefore go beyond checklists and call for structural transformation in policy development ecosystems. Based on the above findings and reflections, we propose the following policy recommendations for structural transformation.

### Policy recommendations

To improve the integration of gender considerations across health policy implementation strategies in Kenya, the following policy recommendations are proposed:

1 Institutionalize gender advocacy in policy formulation.

There is a need to ensure that gender specialists, women’s rights organizations, and civil society actors are systematically included in all stages of policy formulation. For sustainability, this should be formalized through regulatory mandates requiring gender representation in technical working groups and steering committees.

2 Strengthen gender data systems.

The absence of timely, gender-disaggregated data undermines effective policy targeting. The Ministry of Health, in collaboration with the Kenya National Bureau of Statistics (KNBS), should invest in real-time gender-disaggregated surveillance systems. This includes integrating gender metrics into routine health management information systems (HMIS), expanding periodic national health surveys to include gender modules, and ensuring these datasets are publicly accessible for policymaking and academic analysis.

3 Develop gender-specific implementation frameworks.

Policies such as NSP-NCD and NSP-TB must move beyond general equity principles to include actionable, gender-specific interventions. Clear implementation guidelines—including budget lines, timelines, performance indicators, and accountability mechanisms—should accompany all future policies. Counties should be supported to domesticate these frameworks within their integrated development plans (CIDPs).

4 Reframe disease narratives through a gender lens.

The conceptual framing of diseases such as TB and NCDs must shift to explicitly recognize gendered pathways of vulnerability, care-seeking, and outcomes. This includes addressing masculine norms that deter men from accessing care and social norms that place a disproportionate care burden on women. Strategic communication and training for policymakers are necessary to deepen understanding of gender as a structural determinant—not just a binary demographic.

5 Leverage UHC and legal reforms as entry points.

Kenya’s ongoing investment in Universal Health Coverage (UHC) and the constitutional commitment to the Two-Thirds Gender Rule provide strong policy anchors. These should be used to advocate for gender equity in service delivery, resource allocation, and workforce leadership. Legal reviews should be conducted to assess the extent to which sectoral health policies comply with national gender equality laws and the Public Finance Management Act.

6 Build capacity for gender-responsive budgeting.

Policymakers and program managers at national and county levels require capacity strengthening in gender-responsive budgeting (GRB). This includes training on how to design, track, and evaluate budgets that promote gender equity in health. Treasury and Controller of Budget reports should incorporate GRB assessments to promote transparency and resource alignment.

7 Institutionalize monitoring and evaluation (M&E) for gender integration.

Gender integration must be treated as a measurable performance outcome. M&E frameworks should include specific gender indicators, regular audits of policy implementation, and public reporting mechanisms. Independent gender audits should be commissioned periodically to identify gaps and lessons.

8 Mainstream gender in emergency preparedness and response.

The COVID-19 experience revealed the vulnerability of gender equity goals in emergency contexts. Future preparedness frameworks should include protocols for gender analysis, safeguards against gender-based violence, and mechanisms to maintain access to essential health services for all genders during crises.

## Data Availability

The datasets presented in this article are not readily available because the dataset for this study comprises audio recordings and verbatim interview transcripts from key informants—specifically, policymakers and public officials. Due to the nature of their positions and the content of their statements, these individuals are readily identifiable through their voices, titles, institutional affiliations, or specific policy references. Consequently, even with standard anonymization techniques, there remains a significant risk of re-identification. To protect participant confidentiality and comply with ethical standards, access to this dataset is strictly restricted. The data will not be publicly shared and is available only to authorized researchers who: (1) Have obtained prior approval from the relevant ethics review board. (2) Demonstrate a legitimate research purpose aligned with the original study's objectives. (3) Agree to stringent data use agreements that prohibit any attempts at re-identification or unauthorized dissemination. Researchers seeking access must submit a formal request detailing their research objectives, data protection measures, and evidence of ethical approval. Each request will be evaluated on a case-by-case basis, ensuring adherence to all ethical and legal requirements. Requests to access the datasets should be directed to Sylvia Kiwuwa-Muyingo smuyingo@aphrc.org.
