# Supplementary material for: Examining gender as an issue of policy priority: a case study of four Kenyan health policy implementation strategies
Source: Front Public Health. 2025 Jul 31;13:1615792. doi: 10.3389/fpubh.2025.1615792 (PMC12350287; doi:10.3389/fpubh.2025.1615792)
Supplement: Supplementary file 1 [file Data_Sheet_1.docx]

| **Supplementary Table 1. Assessing Policy Document strength on gender consideration** | | | |
| --- | --- | --- | --- |
| **Policy Document** | **Coding** | **Page/section** | **Excerpt** |
| Does the document present Gender-Disaggregated Indicators? | | | |
| KASF II 2020: Kenya AIDS Strategic Framework (KASF) II 2020/21-2024/25 | Strong: KASF II integrates gender-disaggregated data across program design, monitoring, and strategic targeting, especially highlighting disparities among adolescent girls and men. | Page 6 (Executive Summary) | Sex- and age-disaggregated data on HIV and related risk factors were used to inform the design of this Framework. These include higher new infections among adolescent girls and young women (AGYW), and lower treatment coverage among men. |
|  |  | Page 17 (Monitoring & Evaluation) | The KASF II Monitoring and Evaluation Framework includes gender-disaggregated indicators to measure program reach, effectiveness, and equity. For example, coverage rates for HIV testing, PrEP uptake, and ART initiation are disaggregated by sex and age. |
|  |  | Page 18 (Strategic Direction 1) | Efforts will focus on targeted HIV prevention for populations with disproportionately higher new infections, particularly adolescent girls and young women, and men who are less likely to access health services. |
| NSP-NCD  2021: National strategic plan for the prevention and control of non-communicable diseases 2021/22 - 2025/26 | Moderate: The NSP-NCD demonstrates awareness of gender-disaggregated data and includes intentions to collect and report disaggregated indicators, especially under surveillance. However, the absence of specific disaggregated baseline indicators or metrics within the main monitoring framework limits the strength of integration. | Page 16 (Strategic Objectives) | A multisectoral and gender-sensitive approach will be used to improve health literacy… with interventions tailored to the different needs of women and men. |
|  |  | Page 31 (Strategic Objective 3 - Surveillance and Research) | Surveillance and research will consider age and gender disaggregation in data collection and reporting. |
| NSP-TB: National Strategic Plan for Tuberculosis, Leprosy and Lung Health 2019 – 2023 | Strong: The document presents sex-disaggregated baseline indicators, e.g., recognizing higher TB prevalence among men and targeting inequities in financial risk protection. | Page 13 (Foreword) | This NSP recognizes that all human beings have equal rights regardless of their nationality, ethnic origin, sex, race, religion, or any other status and [is] built around core human rights principles… |
|  |  | Page 101 (Outcome Targets) | Tuberculosis approaches and responses at the international level are anchored in international and regional human rights instruments. These laws recognize that all human beings have equal rights regardless of their nationality, ethnic origin, sex, race, religion… |
|  |  | Page 101 (Outcome Targets - Table) | Proportion of households incurring catastrophic costs… DRTB 86% to 43%… Estimated percentage medical insurance coverage among TB patients in Kenya… Estimated direct non-medical costs due to nutrition and food supplements… |
| COVID TTS 2019: Targeted Testing Strategy for Corona Virus Disease 2019 (COVID-19) | Absent: No mention of sex or gender-disaggregated testing data, indicators, or metrics exists anywhere in the COVID-TTS strategy. There are no guidelines or requirements for data disaggregation by gender across reporting templates or outcome tracking mechanisms. | N/A | N/A |
| Does the document cover Institutional Leadership or Roles for Gender Mainstreaming? | | | |
| KASF II 2020: Kenya AIDS Strategic Framework (KASF) II 2020/21-2024/25 | Strong: KASF II explicitly assigns institutional responsibility for gender mainstreaming to multiple actors, including a dedicated Gender TWG and line ministries. | Page 19 (Governance Structures) | The National AIDS Control Council (NACC), in coordination with the Ministry of Health Gender Technical Working Group and the Ministry of Public Service and Gender, will oversee the implementation of gender-responsive programming under KASF II. |
| NSP-NCD  2021: National strategic plan for the prevention and control of non-communicable diseases 2021/22 - 2025/26 | Moderate: There is general acknowledgment of the need for gender-responsive strategies and multi-sectoral implementation. However, no specific institutional roles, such as a gender focal person or coordinating body, are assigned to ensure accountability. The principle is embedded, but without clear operational leadership. | Page 16 (Implementation Principles) | The plan will adopt a life-course approach, incorporating gender-responsive and culturally sensitive strategies implemented through a multi-sectoral framework including the Ministry of Health, County Governments, and other stakeholders. |
| NSP-TB: National Strategic Plan for Tuberculosis, Leprosy and Lung Health 2019 – 2023 | Weak: There is no designated institutional leadership, focal point, or coordination mechanism for gender integration in the NSP-TB. Mentions of "Ministry of Labour" and general calls to stakeholders exist, but these are not institutionally tied to gender leadership. | N/A | N/A |
| COVID TTS 2019: Targeted Testing Strategy for Corona Virus Disease 2019 (COVID-19) | Absent: No institutional roles (e.g., gender focal points, technical working groups) are assigned or mentioned in relation to gender equity. Coordination roles focus solely on testing, laboratory oversight, and county-level scale-up. | N/A | N/A |
| Does the document Frame Gender as a Structural Driver of Health Inequity? | | | |
| KASF II 2020: Kenya AIDS Strategic Framework (KASF) II 2020/21-2024/25 | Moderate to Weak: While KASF II acknowledges gender as a structural driver, the depth of analysis is uneven. The strongest statements appear in the strategy preambles, but do not consistently translate into structural interventions. | Page 18 (Strategic Direction 1) | KASF II will support gender transformative approaches that address power relations, norms, and systemic disadvantages that sustain the epidemic. |
| NSP-NCD  2021: National strategic plan for the prevention and control of non-communicable diseases 2021/22 - 2025/26 | Weak: While the plan references gender sensitivity, it does not interrogate the structural dimensions of gender—such as power relations, normative roles, or systemic inequities—that contribute to differential NCD risk or care outcomes. The framing remains superficial and does not link gender to systemic disadvantage. | Page 16 (Guiding Principles) | Interventions shall be culturally acceptable, gender-sensitive, and equitable across age groups. |
| NSP-TB: National Strategic Plan for Tuberculosis, Leprosy and Lung Health 2019 – 2023 | Weak: Although the document recognizes gender norms as barriers, the discussion is surface-level and does not engage power, patriarchy, or systemic discrimination. Gender is acknowledged but not structurally problematized. | Excerpt – Page 102 (Gender Acknowledgement) | This NSP acknowledges that gender inequalities can impact health risks, health seeking behaviour and responses from health systems, which lead to poorer outcomes… responsive programming, which considers the prevailing gender norms or undertakes gender transformative programming, so as to mitigate harmful gender norms… |
| COVID TTS 2019: Targeted Testing Strategy for Corona Virus Disease 2019 (COVID-19) | Absent: The strategy does not acknowledge how gender roles, norms, or inequalities might affect exposure, access to testing, or case management. There is no framing of gender as a structural factor - neither explicitly nor implicitly - in the policy. | N/A | N/A |

| **Supplementary Table 2.** **Examining Factors Influencing Gender as an Issue of Policy Priority Data Extraction Tool for Policy Document** | | | | | | | | | | | |
| --- | --- | --- | --- | --- | --- | --- | --- | --- | --- | --- | --- |
|  | **Actors** | | | **Ideas** | | **Policy Environment** | | | **Issue Characteristics** | | |
| **Guiding Questions for Document Analysis** | - Are key institutions or individuals named as responsible for gender integration? - Are interagency or multisectoral collaborations mentioned? | | | - How is gender discussed or defined? - Is gender framed as an equity, efficiency, human rights, or population issue? | | - Does the policy reference national or international commitments (e.g., CEDAW, SDGs, Constitution)?  - Are there links to existing gender policies? | | | - Is gender inequality presented as a measurable or solvable problem?  - Are there indicators, targets, or action plans related to gender? | | |
| **Indicators/Variables to Extract** | Named agencies/focal points | Gender-specific working groups | External partners (donors, NGOs) | Framing language (rights-based, technical, political) | Depth of conceptualization | Gender vs. sex usage | Referenced frameworks (e.g., Kenya Constitution, Gender Policy, SDGs) | Timeline context (e.g., election years) | Gender-disaggregated indicators | Framing of gender as a driver of inequity | Intervention strategies |
| **KASF II 2020** |  |  |  |  |  |  |  |  |  |  |  |
| **NSP-NCD  2021** |  |  |  |  |  |  |  |  |  |  |  |
| **NSP-TB** |  |  |  |  |  |  |  |  |  |  |  |
| **COVID TTS 2019** |  |  |  |  |  |  |  |  |  |  |  |

| **Supplementary Table 3.** **Examining Factors Influencing Gender as an Issue of Policy Priority Data Extraction Tool for Key informant interviews Document** | | | | | | | | | | | | | |
| --- | --- | --- | --- | --- | --- | --- | --- | --- | --- | --- | --- | --- | --- |
| **Shiffman & Smith Domain** | **Actors** | | | | **Ideas** | | | **Policy Environment** | | | **Issue Characteristics** | | |
| **Guiding Questions for Interviews** | - Who were the key individuals, institutions, or networks that influenced policy development? - What roles did bureaucrats, politicians, donors, or civil society play in advancing (or stalling) gender issues? | | | | - How was gender framed during the policy process? - What narratives or discourses were dominant (e.g., gender as a rights issue, a technical fix, or a political risk)? | | | - What was the broader political and institutional climate during policy formulation? - Were there enabling or constraining laws, global frameworks, or political events? | | | - What evidence was used to define gender as a priority or not? - Was gender discussed in terms of severity, solvability, or urgency? | | |
| **Key Variables/Indicators** | Named actors | Institutional affiliations | Actor influence level | Gender advocacy role | Dominant gender frames | Competing ideas | References to equity, rights, burden, or norms | Enabling/constraining legislation | Window of opportunity | Influence of donors/global bodies | Use of disaggregated data | Framing of burden or impact | Mention of interventions or outcomes |
| **KASF II 2020** |  |  |  |  |  |  |  |  |  |  |  |  |  |
| **NSP-NCD  2021** |  |  |  |  |  |  |  |  |  |  |  |  |  |
| **NSP-TB** |  |  |  |  |  |  |  |  |  |  |  |  |  |
| **COVID TTS 2019** |  |  |  |  |  |  |  |  |  |  |  |  |  |

**Gendered health pathways project: Workstream 3 - Policy analysis**

**Key informant interviews: Question guide**

**QUESTIONS**

| **Opening questions** | |
| --- | --- |
| ***Need not cover all or any of these questions and may include others. The aim is to put the informant at ease and to make sure they understand which policy we are talking about and that we are talking about formulation of the policy (getting the words on paper).***   1. What was your role in relation to the development, adoption or implementation of [name of health policy or health policy area] in Kenya? 2. What is your view of the effectiveness of this policy at achieving [state aims of the policy]? 3. We found that the policy was [gender-responsive or gender-blind or gender-specific], specifically due to [state evidence from the policy justifying this scoring].    1. Does this concur with your understanding of the gender-responsiveness of the policy? 4. What is your general understanding of why gender transformative actions( (such as promotion of gender equality, transform harmful gender norms, roles, and relations, addressing the causes of gender-based health inequities etc.)) was/was not taken into account? | |
| Main questions | |
| 1. We are using a framework to understand why policy takes the shape that it does. Specifically, we are using a framework on agenda setting to understand why gender was or was not incorporated into [name of health policy]. The framework suggests that it is likely because of [briefly summarize four categories of framework]. We want you to reflect, based on your experience, on a few of the areas in the framework.   *Proceed by focusing on 2-3 selected categories + 2-3 selected factors within each category.* | |
| 1. **The power of the ACTORS involved in the issue** | 1. Who were the key actors, if any, involved in, or with an influence on, promoting the recognition of gender in the formulation of this policy? (**Prompt**: Key actors may include prominent leaders of non-governmental organizations, elected or appointed high-level government officials and /or other senior bureaucrats, professional associations, private sector, bilateral donors, members of UN agencies other international organizations, and academics) 2. Was there anyone involved in policy formulation who led the push for gender to be recognized?    1. If so, who were they?    2. Were they included in the policy process? Were they respected by, and capable of uniting, the policy community? 3. Which actors, if any, *resisted* the recognition of gender in policy agenda-setting, formulation and development?    1. If so, who were they?    2. Were they respected by, and capable of uniting, the policy community? 4. Was there a loose/informal community working on gender equality in health in Kenya?    1. If so, who were they?    2. What was the degree of cohesion among this policy community - i.e. the extent to which they were united on basic issues such as how gender should be addressed?    3. Were they included in the policy formulation process and why/why not? 5. Were there any strong guiding institutions - i.e, organizations or coordinating mechanisms with a mandate to lead attempts to make the policy gender-responsive? - **Prompt**: e.g. the local office of UN Women (or other example of a domestic agency with a gender mandate)    1. If so, who were they? 6. Were there grassroots organizations/civil society mobilized to push for international or national attention to gender in [name of health area]?    1. If so, who were they?    2. To what extent was there cohesion among these groups and between them and the global gender community?    3. How were they organized (e.g through protest, lobbying, media engagement)? 7. What was the role of the media in either promoting or suppressing gender as an issue? 8. What was the gender distribution of the key decision-makers for formulation of this policy? |
| 1. **The power of the IDEAS used by actors to portray the issue** | 1. In your view, is there a policy community that has an *internally* shared understanding of gender inequality and how it relates to health—and how gender should be reflected in health policies?    1. If so, how do they frame gender and how do they try to sell it to health policy makers?    2. Which individuals/groups were primarily responsible for developing this shared framing? 2. Was a frame of gender in health developed that resonated *externally* to move essential individuals and organizations to action to ensure that gender was reflected in the policy? E.g. political leaders who control resources that initiatives need?    1. If so, what was the frame?    2. Which individuals/groups were primarily responsible for developing this shared framing? 3. Did specific political/social/cultural norms/values in the country influence how gender was framed in the policy under consideration?    1. If so, what were these norms/values and how did they shape the framing? |
| 1. **The nature of the POLITICAL CONTEXTS in which actors operated** | - 1. Did public opinion/recognition of the importance of gender equality at the time the policy was being developed act as an enabler or barrier to developing a gender-responsive policy?      1. If so, how? Did the media play a role in shaping public recognition of the issue?   2. Was there a diversity of political ideologies/political backgrounds among the policymakers or was there a dominant political ideology/ political background?      1. What were the ideologies/backgrounds?      2. Did this impact policy formulation and, if so, how? (i.e. did political/cultural values prevent the policymakers from going further on gender?)   3. Was there a global governance structure - i.e., a set of norms and the institutions that negotiate and enforce these norms - for gender to be recognized in [name of health area]? (**Prompt**: this could include international treaties, laws and declarations)      1. If so, what was this structure?      2. To what degree did it provide a platform for effective collective action on gender? |
| 1. **CHARACTERISTICS OF THE ISSUE** | - 1. Were there clear measures of the severity of gender differences (e.g. in disease risk factors and outcomes) in [name of health area] and metrics and monitoring platforms that could be used to monitor progress?      1. If so, what were these metrics and platforms?   2. Was there evidence of the severity of gender inequity in health outcomes in [name of health area] relative to other problems, indicated by objective measures (e.g. mortality levels)?      1. Which, if any, other health issues were prioritized for a gender lens due to their real or perceived severity?   3. Were there available means of addressing gender inequities that were clearly explained, cost effective, backed by scientific evidence, simple to implement, and cost-effective?      1. What were these interventions?      2. Who proposed/pushed for them to be implemented? |
| **Closing questions** | |
| 1. **ESSENTIAL: Overall, of the factors we have discussed, do any standout as particularly important in determining why gender was/wasn’t recognized?** 2. Are there any factors that you think we have missed? 3. Apart from the strategy talking about disaggregation of data based on sex, did you consider gender transformative actions (such as promotion of gender equality, transform harmful gender norms, roles, and relations, addressing the causes of gender-based health inequities etc.). 4. If no in 8, why not. If yes, which actions? Why were these actions not included on the strategy? 5. Are there any other points that you would like to make in relation to the attention to gender in this policy that we haven’t discussed? 6. Are there any other people/departments/organizations that you think we would benefit from talking to? [consider whether appropriate, dependent on the interviewee, to share list of participants here] 7. Thank the informant for their time, explain next steps of the study and any follow up they can expect from us and by what date (e.g. we will share the draft report with them). Ask whether they would be interested in commenting on draft report. | |
